# Multi-omics analysis of fecal microbiota transplantation’s impact on functional constipation and comorbid depression and anxiety

**DOI:** 10.1186/s12866-023-03123-1

**Published:** 2023-12-07

**Authors:** Chuanli Yang, Tianjiao Hu, Xin Xue, Xiaohu Su, Xuan Zhang, Yunhe Fan, Xiaobing Shen, Xiushan Dong

**Affiliations:** 1 Key Laboratory of Environmental Medical Engineering and Education Ministry, School of Public Health, Southeast University, Nanjing, Jiangsu China; 2 Department of Preventive Medicine, School of Public Health, Southeast University, Nanjing, China; 3 Department of General Surgery, Tongji Shanxi Hospital, Shanxi Bethune Hospital, Shanxi Academy of Medical Sciences, Third Hospital of Shanxi Medical University, Taiyuan, China

**Keywords:** Constipation, Depression and anxiety, Fecal microbiota transplantation, Metagenomics, Metabolomics, 5-hydroxytryptamine

## Abstract

**Background:**

Depression and anxiety are common comorbid diseases of constipation. Fecal microbiota transplantation (FMT) significantly relieves gastrointestinal-related symptoms, but its impact on psychiatric symptoms remains uncharted.

**Methods:**

We collected fecal and serum samples before and after FMT from 4 functional constipation patients with psychiatric symptoms and corresponding donor stool samples. We categorized the samples into two groups: before FMT (Fb) and after FMT (Fa). Parameters associated with constipation, depression, and anxiety symptoms were evaluated. Metagenomics and targeted neurotransmitter metabolomics were performed to investigate the gut microbiota and metabolites. 5-hydroxytryptamine (5-HT) biosynthesis was detected in patients’ fecal supernatants exposed to the QGP-1 cell model *in vitro*.

**Results:**

Our study demonstrated that patient’s constipation, depression, and anxiety were improved after FMT intervention. At the genus level, relative abundance of *g_Bacteroides* and *g_Klebsiella* decreased in the Fa group, while *g_Lactobacillus*, and *g_Selenomonas* content increased in the same group. These observations suggest a potential involvement of these genera in the pathogenesis of constipation with psychiatric symptoms. Metabolomics analysis showed that FMT intervention decreased serum 5-HT levels. Additionally, we found that species, including *s_Klebsiella sp. 1_1_55*, *s_Odoribacter splanchnicus*, and *s_Ruminococcus gnavus CAG:126*, were positively correlated with 5-HT levels. In contrast, *s_Acetobacterium bakii*, *s_Enterococcus hermanniensis*, *s_Prevotella falsenii*, *s_Propionispira arboris*, *s_Schwartzia succinivorans*, *s_Selenomonas artemidis*, and *s_Selenomonas sp. FC4001* were negatively correlated with 5-HT levels. Furthermore, we observed that patients’ fecal supernatants increased 5-HT biosynthesis in QGP-1 cells.

**Conclusion:**

FMT can relieve patients’ constipation, depression, and anxiety symptoms by reshaping gut microbiota. The 5-HT level was associated with an altered abundance of specific bacteria or metabolites. This study provides specific evidence for FMT intervention in constipation patients with psychiatric symptoms.

**Supplementary Information:**

The online version contains supplementary material available at 10.1186/s12866-023-03123-1.

## Introduction

Functional constipation (FC) is a prevalent functional bowel disorder more commonly observed in women, elderly individuals, and those of lower socioeconomic status [1–3]. FC severely impacts the quality of life and represents a tremendous healthcare burden. Depression is a mood disorder characterized by persistent low mood, lack of motivation, loss of pleasure, and body dysfunction. Numerous studies have established a link between depression and gastrointestinal diseases. A large-scale meta-analysis has demonstrated that anxiety, depression, and constipation often coexist as comorbidities across various types of irritable bowel syndrome (IBS), including the subtype dominated by constipation [4]. In the general population, both IBS and inflammatory bowel disease (IBD) are associated with the incidence rate and severity of depression [5–7]. Up to 50% of IBS patients and 15–25% of IBD patients meet the clinical diagnostic criteria for depression [8].

Although the pathogenesis of constipation comorbid depression and, anxiety remains elusive, currently recognized theories include the microbiome-mediated bidirectional communication model or the so-called “brain-gut-microbiome axis”. Jennifer et al. confirmed that IBS sub-types based on intestinal microbiota were associated with brain structural changes [9]. In addition, Labus et al. demonstrated that intestinal microbial metabolites may act as intermediaries for disease-related brain structural changes in IBS patients [10], suggesting correlations between gut microbiota and gastrointestinal diseases and brain function. In healthy women, consumption of fermented milk product with probiotics for four weeks altered the gut microbiota composition [11]. In addition, functional magnetic resonance imaging (MRI) showed that the central processing brain regions of the participants, which control emotion and sensation, were activated [11]. A randomized, double-blind, placebo-controlled trial demonstrated that fermented milk containing *Lacticaseibacillus paracasei* strain Shirota could relieve constipation and depression symptoms in patients and reduce the level of bacteria related to mental diseases, such as *Rikenellaceae_RC9_gut_group*, *Sutterella*, and *Oscillibacter* [12]. These above studies suggested that the gut microbiota and brain interaction may be involved in the progression and development of constipation in patients with mental disease.

Currently, several potential approaches are available to rebalance the intestinal microbiota ecosystem, such as diet, lifestyle, antibiotics, probiotics, prebiotics, synbiotics, and fecal microbiota transplantation (FMT) [13]. However, FMT is an effective treatment that drastically alters the gut microbial profile. FMT contains thousands of species compared to probiotics, which only comprise a few specific bacterial species. A myriad of studies have shown that FMT is effective in the treatment of gastrointestinal dysfunction diseases, such as chronic constipation [14], IBS [15], and IBD [16]. Moreover, FMT may also be effective in relieving some neurological disorders. Langgartner et al. showed that multiple FMT from stress-free mice to animals exposed to chronic mental stress reduced anxiety and depression-like symptoms in recipients [17]. In addition, Yang et al. demonstrated that mice receiving FMT from anhedonic rats improved their depressive-like symptoms [18]. In a population study, Mizuno et al. found that the psychiatric state of patients with IBS was significantly relieved one month after healthy individuals’ gut microbiota transplantation [19]. Similarly, Huang et al. noticed that the HAMD and HAMA scores of IBS subjects significantly improved at 1 and 3 months after FMT from a healthy donor [15]. In addition, Kurokawa et al. found a correlation between increased microbiome diversity and improved depression scores after FMT treatment [20] .The above studies indicate that FMT can alleviate both gastrointestinal and psychiatric symptoms. However, the mechanism of FMT in the comorbidity of gastrointestinal and psychiatric diseases remains unclear.

The primary purpose of the present study was to explore the effect and potential mechanisms of FMT on constipation in patients experiencing the symptoms of depression and anxiety. Intestinal and psychiatric symptoms were evaluated before and after FMT. Furthermore, gut microbiota and the metabolic profile of neurotransmitters were also determined by metagenomic sequencing and targeted metabolomics analysis, respectively. This study is expected to provide evidence for the effectiveness of FMT treatment and reveal the relationship between gut microbiota and microbiota-derived metabolites in constipated patients with psychiatric symptoms.

## Materials and methods

### Participants

The investigation was conducted at the Shanxi Bethune Hospital in Taiyuan, Shanxi, China. 4 patients diagnosed as FC with depression and anxiety were recruited for FMT treatment. The enrolment criteria were as follows: age ≥ 18 years; FC was assessed and diagnosed according to the Rome IV Diagnostic Criteria [21] persistent constipation symptoms for at least one year; depression and anxiety were evaluated and diagnosed with the DSM-V criteria; and depressive symptoms lasted more than 12 months. Exclusion criteria were as follows: organic or neurological constipation; diagnosed with other mental diseases; pregnant or lactating women; diagnosed with IBD, malignant tumors, or gastrointestinal surgery; abnormal thyroid function; use of antidepressants, probiotics, prebiotics, and antibiotics within two weeks before the study; and subjects in any other studies. Among donors with good personal habits (≥ 18 years of age, BMI: 18.5–23.9 kg/m^2^), donors were further screened using serology and stool screening for common enteric and viral pathogens. Donors were excluded if they used proton pump inhibitors and antibiotics six months before FMT donation (donors were selected by Shanghai WellBody Biotechnology Co., Ltd. Shanghai, China). Four FC patients with depression and anxiety underwent FMT from March 2021 to June 2021 were included. This study was approved by the Ethics Committee of Shanxi Bethune Hospital (No. XYLL-2019-124), and the study was in accordance with national laws and the Declaration of Helsinki.

### Data collection

This study employed a single– centre, open-label, nonrandomized approach to investigate the effect of FMT on FC patients with depression and anxiety. The Bristol stool form scale (BSFS), Bowel Function Index (BFI), Knowles Eccersley Scott Symptom (KESS), and Patient Assessment of Constipation Quality of Life (PAC-QOL) were used to assess constipation parameters. The psychological symptoms of patients were evaluated by experienced psychiatrists or psychologists using the Hamilton Depression Rating Scale (HAMD). A HAMD score ≥ 8 is considered to indicate depression. In addition, the Self-Rating Depression Scale (SDS) and Self-Rating Anxiety Scale (SAS) were also used to assess depression and anxiety symptoms. All scales were evaluated at baseline and four weeks after FMT.

### FMT procedure and sample collection

Donors were required to collect feces at Shanghai WellBody Biotechnology Co., Ltd. (Shanghai, China, www.wellbodybio.com). Collected donor fecal samples were weighed, homogenized, and mixed with saline in a 1:5 ratio. After multi-stage filtration, the samples were then dispensed into centrifuge tubes and resuspended by low temperature centrifugation to precipitate the microbiota. Depending on the gradient of the filter aperture, food residues, and impurities are removed and all microbiota were collected, containing metabolites of all types of flora. Collected samples were aliquoted (50 mL/tube) and stored at -80 °C. The feces were thawed at 4 °C on the day of FMT. On days 1–6, 100 mL of fresh feces was transplanted to patients through a nasointestinal tube once daily. A total of 2 courses of treatment were conducted, each course lasting 36 days, with the FMT intervention for the first 6 days and then no intervention for 30 days. The nasointestinal tube was installed in the proximal jejunum through an endoscope. Fecal samples of donors and patients at baseline and four weeks after FMT were collected for metagenomic analysis. Serum samples of patients at baseline and 4 weeks after FMT treatment were collected for targeted metabonomic analysis.

### Metagenomic sequencing and analysis

Methods of fecal DNA isolation and metagenomic sequencing analysis were reported in our previous research [22]. The gut microbiome was analyzed via metagenomic shotgun sequencing. Genomic DNA was extracted from fecal samples and assessed for quality. Certified DNA samples were fragmented into 350 bp fragments, and the library was prepared through a series of steps, including end repair, A-tail addition, adapter ligation, purification, and amplification. Sequencing was conducted on an Illumina PE150 platform (Shanghai Biotree biomedical technology). The raw sequencing data underwent rigorous quality control to obtain high-quality data. These refined data were assembled via metagenome analysis, with gene prediction facilitated by the widely-used software, MetaGeneMark. The analyzed data were cross-referenced with the MicroNR library to obtain species annotation information of UniGene. Furthermore, we performed Kyoto Encyclopedia of Genes and Genomes (KEGG) metabolic pathway function annotation and abundance analysis. Nonmetric multidimensional scaling (NMDS), and Hierarchical cluster analysis were conducted based on species abundance. The core-pan gene rarefaction curve is a tool used to analyze microbiomes. This curve is generated by randomly selecting varying numbers of samples and calculating the relationship between core genes and pan genes observed at different sample quantities. The core-pan gene rarefaction curve is typically plotted with the number of samples on the X axis, and the counts of core genes and all genes on the Y axis. We calculated Chao1, observed species and Shannon’s index to find information on species richness and evenness. NMDS analysis was used to investigate the comparison of microbial β diversity among the three groups.

### Targeted metabonomic analysis based on UHPLC–MS/MS

Methods of serum targeted metabonomic analysis were reported in our previous research [22]. We performed targeted metabolomic analysis on serum samples before and after FMT. Each 20 µL sample was mixed with 80 µL pre-cooled extract solvent (acetonitrile with 0.1% formic acid). After vortexing, sonication, and overnight settling at − 40℃, the samples were centrifuged. The supernatant (80 µL) was combined with 40 µL of 100 mM carbonate solution and 40 µLof 2% benzoyl chloride acetonitrile solution for a 30-minute incubation. Following the addition of 10 µL of internal standard, the samples were centrifuged, and 40 µLof supernatant was mixed with 20 µL H_2_O. These samples were subjected to ultra-high-performance liquid chromatography coupled with mass spectrometry (UHPLC-MS/MS) analysis, which followed established procedures. UHPLC separations used an ExionLC system with a Waters ACQUITY UPLC HSS T3 column. The data were acquired with an AB Sciex QTrap 6,500 + mass spectrometer and analyzed using Skyline software. Orthogonal partial least squares discriminant analysis (OPLS-DA) analysis is a multivariate statistical method commonly used in metabolomics data analysis for identifying differential metabolites between different groups. In the current study, we utilized Simca software (version 15.0.2) for modeling. One predictive principal component and one orthogonal principal component were used. Cross-validation was performed with a 7-fold approach, and existing data were employed as the training set for modeling. In addition, the volcano plot was prepared by applying Simca software. The bubble plot in the pathway analysis was taken from the MetaboAnalyst web (https://www.metaboanalyst.ca/).

### Cell culture

Human pancreatic endocrine QGP-1 cell line was purchased from COBIOER Biological Company (Nanjing, China). Cells were cultured in RPMI 1640, supplemented with 10% FBS (BI, Israel), and incubated at 37℃ with 5% CO_2_.

### Western blot (WB) analysis

Total cellular lysates were obtained by collecting cells in RIPA buffer (Beyotime Biotechnology, Shanghai, China) on ice. An equivalent amount of sample protein was loaded onto SDS-PAGE, and then transferred onto PVDF membrane (Millipore, USA). The membranes were incubated at 4 °C overnight with primary antibodies—anti-TPH-1 (1:1000, Affinity Biosciences, DF6465) and anti-GAPDH (1:10,000, ABclonal, AC002). Subsequently, the membranes were incubated for an hour with secondary antibodies conjugated with horseradish peroxidase, and the subsequent visualization was performed using an imaging system (Azure C300, USA) along with an enhanced Chemiluminescence Kit (Epizyme Biotech, China).

**Table 1:** Sociodemographic information of the study subject

Subject	Age (years)	Gender (F/M)	Body mass index (kg/m^2^)	Years with diagnosed constipation (mean ± SD)	Years with diagnosed depression (mean ± SD)
Patient 1	68	female	28.72	17	4
Patient 2	56	female	21.51	20	25
Patient 3	66	female	23.5	10	2
Patient 4	66	female	21.34	3	3
Total (Mean ± SD)	64.00 ± 5.41	NA	23.77 ± 3.44	12.25 ± 8.02	7.00 ± 8.72
Donor 1	23	female	21.72		
Donor 2	31	male	20.06		
Donor 3	24	male	22.48		
Donor 4	31	female	20.68		
Total (Mean ± SD)	27.25 ± 4.35	NA	21.24 ± 1.08		

**Table 2:** The effects of the FMT on constipated and psychiatric symptoms

Item	Fb	Fa	*P* value
Bristol stool scale (BSS)	II (all)	IV (all)	
Bowel functional index (BFI)	273.75 ± 30.38	116.25 ± 110.71	0.034
Knowles-Eccersley-Scott-Symptom (KESS)	28.25 ± 3.30	12.25 ± 5.74	0.003
Hamilton Rating Scale for Depression (HAMD)	38.50 ± 3.11	7.25 ± 1.26	< 0.001
Hamilton Rating Scale for Anxiety (HAMA)	39.00 ± 9.93	13.75 ± 6.75	0.006
Self-Rating Anxiety Scale (SAS)	54.75 ± 5.91	38.25 ± 8.66	0.020
Self-Rating Depression Scale (SDS)	66.00 ± 4.83	48.75 ± 12.97	0.047
Patient Assessment of Constipation Quality of Life (PAC-QOL)	103.50 ± 11.12	64.00 ± 23.39	0.023

### Quantitative real-time polymerase chain reaction (qRT-PCR)

Total RNA was isolated from QGP-1 cells by RNA-easy isolation reagent (Vazyme, China) and reversely transcribed into cDNA with HiScript Reverse Transcriptase Kit (Vazyme, China) according to the manufacturer’s instruction. The 2^−ΔΔCt^ method was used to evaluate the relative expression.

**Fig. 1:**
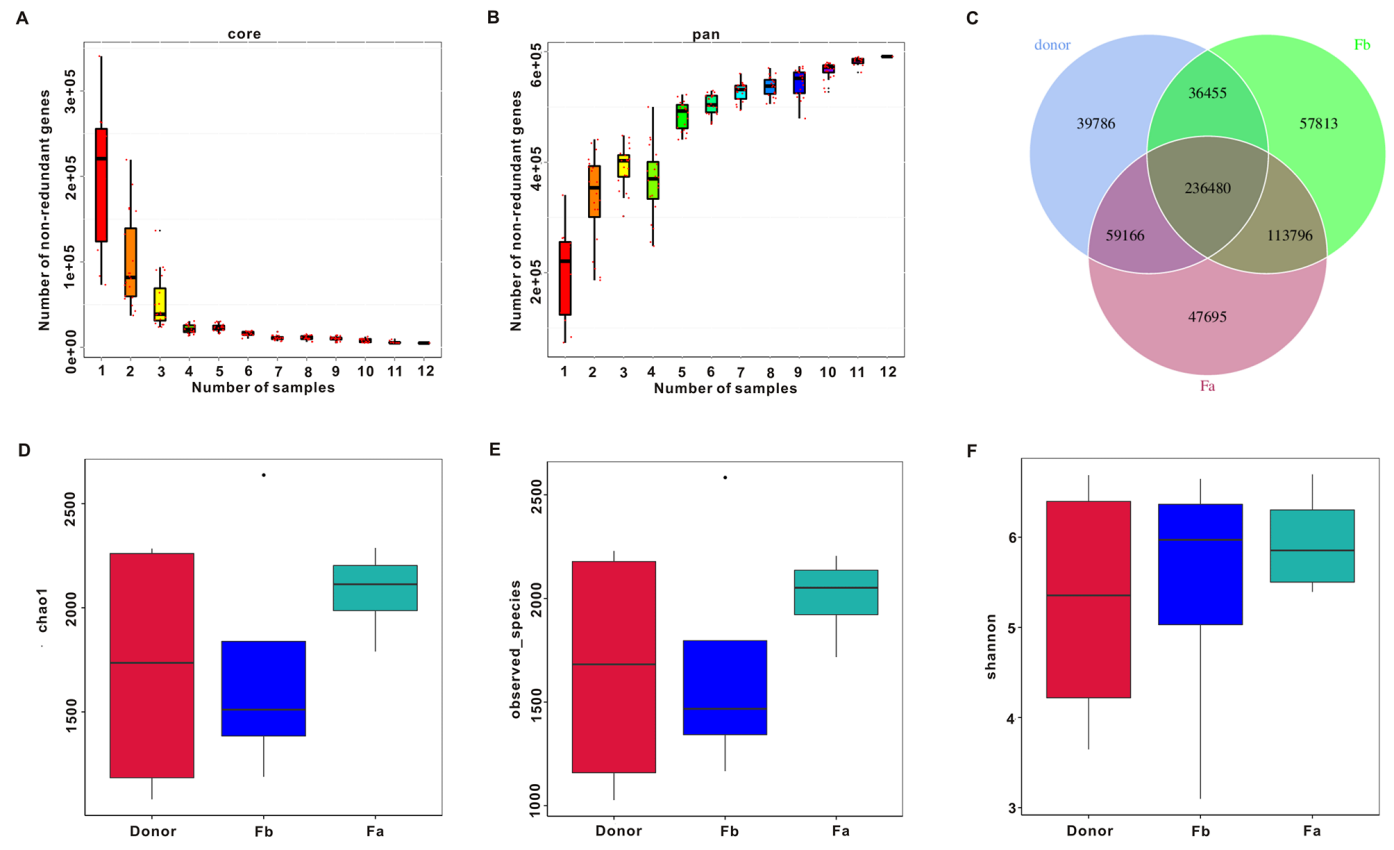
Gene expression, species abundance and diversity of the gut microbiota. Rarefaction curves of (**A**) core genes and (**B**) pan genes. (**C**) Venn diagram of the observed genes. Boxplot of chao1 (**D**) observed species (**E**) and Shannon index (**F**)

### Cell counting kit-8 (CCK-8) proliferation assay

QGP-1 cells were seeded in 96-well plate at a density of 20,000 cells/well. Once the cells adhered, they were treated with fecal supernatant (fecal supernatant: medium = 1:5). Subsequently, 10 µL CCK-8 solution was added to each well, and incubated for 2 h. The optical density of the cells was measured using microplates at 450 nm.

### Enzyme-linked immunosorbent assay (ELISA)

For 5-HT analysis, the QGP-1 cell culture medium supernatant was collected. The measurements were performed using 5-HT ELISA Kit (Elabscience, E-EL-0033c) according to the manufacturer’s protocols.

**Fig. 2:**
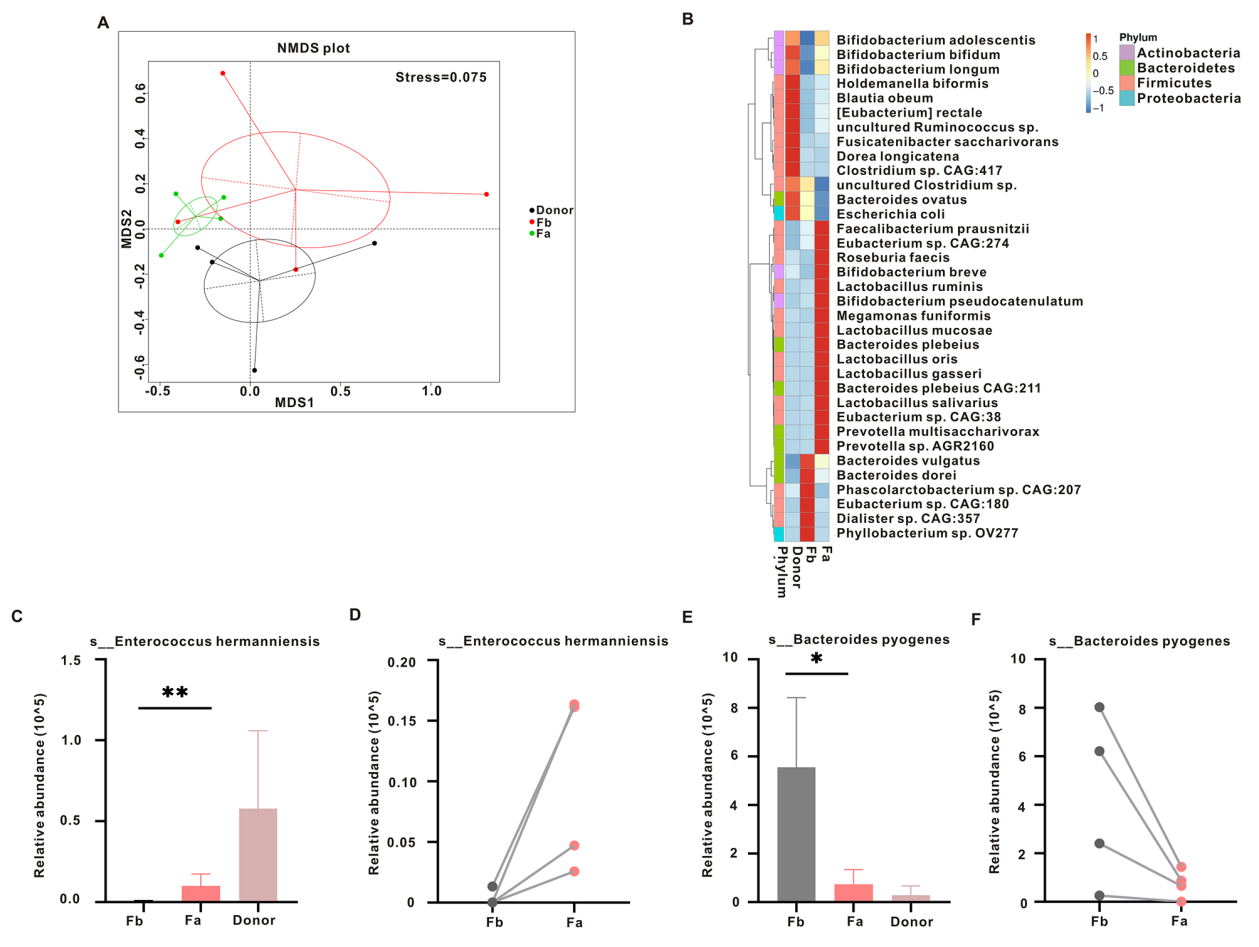
The shift in gut microbiota before and after FMT based on metagenomic sequencing data. (**A**) Nonmetric multidimensional scaling (NMDS) of the fecal microbiota for donors and before and after FMT. (**B**) Relative abundance of species (top 10) between the three groups. Relative abundance of *s_Bifidobacterium longum* (**C**) and *s_Enterococcus hermanniensis* (**E**) among the three groups (Kruskal-Wallis test). Relative abundance of *s_Bifidobacterium longum* (**D**) and *s_Enterococcus hermanniensis* (**F**) before FMT compared to after FMT. *P < 0.05, **P < 0.01

### Statistical analysis

Statistical analysis was performed using SPSS 22.0 software. The BFI, KESS, PAC-QOL, HAMA, HAMD, SAS, and SDS scores are presented as the mean ± standard deviation (SD). Differences were determined using Student’s *t*-test if the data conformed to a normal distribution, otherwise, Wilcoxon rank-sum test was used. We calculated the Spearman’s correlation coefficients for metabolites and significant bacteria in each sample to generate a correlation matrix, then selected the Euclidean distance for hierarchical clustering analysis, and finally generated a heat map. Receiver operating characteristic (ROC) working characteristic curve analysis was used to detect serum 5-HT application value by judging the before and after FMT treatment. Area under the curve (AUC) > 0.75 was considered good accuracy [23]. *P* values below 0.05 were considered to represent a significant difference.

**Table 3:** The top 15 different species with up-regulated and down-regulated expression in threes groups

Genus	Species	mean D	mean Fb	mean Fa	log2 fold change (Fa/Fb)	*P* value
g_Bacteroides	s_Bacteroides faecichinchillae	0.000003	0.000063	0.000007	-3.147	0.0227
	s_Bacteroides pyogenes	0.000003	0.000042	0.000007	-2.514	0.0277
	s_Bacteroides stercorirosoris	0.000000	0.000043	0.000006	-2.880	0.0142
g_Odoribacter	s_Odoribacter splanchnicus	0.000153	0.000501	0.000090	-2.479	0.0104
g_Prevotella	s_Prevotella falsenii	0.000000	0.000000	0.000038	6.851	0.0440
g_Bacillus	s_Bacillus sp. UNC41MFS5	0.000001	0.000002	0.000000	-7.134	0.0467
	s_Bacillus wiedmannii	0.000005	0.000000	0.000005	5.472	0.0490
g_Listeria	s_Listeria monocytogenes	0.000012	0.000001	0.000000	-2.574	0.0292
g_Brevibacillus	s_Brevibacillus laterosporus	0.000003	0.000000	0.000001	5.234	0.0276
g_Paenibacillus	s_Paenibacillus algorifonticola	0.000001	0.000002	0.000000	-2.548	0.0172
g_Enterococcus	s_Enterococcus hermanniensis	0.000006	0.000000	0.000001	4.912	0.0078
g_Lactobacillus	s_Lactobacillus acidophilus	0.000000	0.000000	0.000005	5.794	0.0203
	s_Lactobacillus coleohominis	0.000000	0.000000	0.000024	7.771	0.0459
	s_Lactobacillus gallinarum	0.000000	0.000001	0.000038	4.981	0.0312
	s_Lactobacillus plantarum	0.000000	0.000001	0.000040	4.924	0.0413
g_Clostridium	s_Clostridium formicaceticum	0.000001	0.000000	0.000008	8.241	0.0095
g_Natronincola	s_Natronincola peptidivorans	0.000001	0.000001	0.000000	-3.231	0.0162
g_Acetobacterium	s_Acetobacterium bakii	0.000004	0.000000	0.000004	4.673	0.0113
g_Blautia	s_[Ruminococcus] gnavus	0.003542	0.002449	0.000387	-2.660	0.0025
g_Pseudobutyrivibrio	s_Pseudobutyrivibrio sp. UC1225	0.000000	0.000002	0.000000	-5.699	0.0091
g_Unclassified	s_Lachnospiraceae bacterium 2_1_58FAA	0.000085	0.000080	0.000016	-2.301	0.0309
g_Ruminococcus	s_Ruminococcus gnavus CAG:126	0.001085	0.000640	0.000036	-4.147	0.0041
g_Unclassified	s_Clostridiales bacterium VE202-13	0.000008	0.000000	0.000007	5.853	0.0184
g_Propionispira	s_Propionispira arboris	0.000002	0.000000	0.000006	4.696	0.0389
g_Schwartzia	s_Schwartzia succinivorans	0.000002	0.000000	0.000012	6.338	0.0295
g_Selenomonas	s_Selenomonas artemidis	0.000001	0.000000	0.000013	5.442	0.0063
	s_Selenomonas sp. FC4001	0.000006	0.000000	0.000006	6.598	0.0195
g_Klebsiella	s_Klebsiella pneumoniae	0.000442	0.000305	0.000014	-4.415	0.0037
	s_Klebsiella sp. 1_1_55	0.000026	0.000012	0.000002	-2.543	0.0246
	s_Klebsiella sp. HMSC16C06	0.000001	0.000001	0.000000	-4.526	0.0402

*P* < 0.05 represent the comparison Fb vs. Fa.

## Results

### Patient sociodemographic characteristics

The clinical features of patients and donors are displayed in Table 1. The average age was 64.00 ± 5.41 years in patients and 27.25 ± 4.35 years in donors. The body mass index of the patients and donors were 23.77 ± 3.44 and 21.24 ± 1.08 kg/m^2^, respectively. The average duration of constipation and depression were 12.25 ± 8.02 years and 7.00 ± 8.72 years, respectively.

**Fig. 3:**
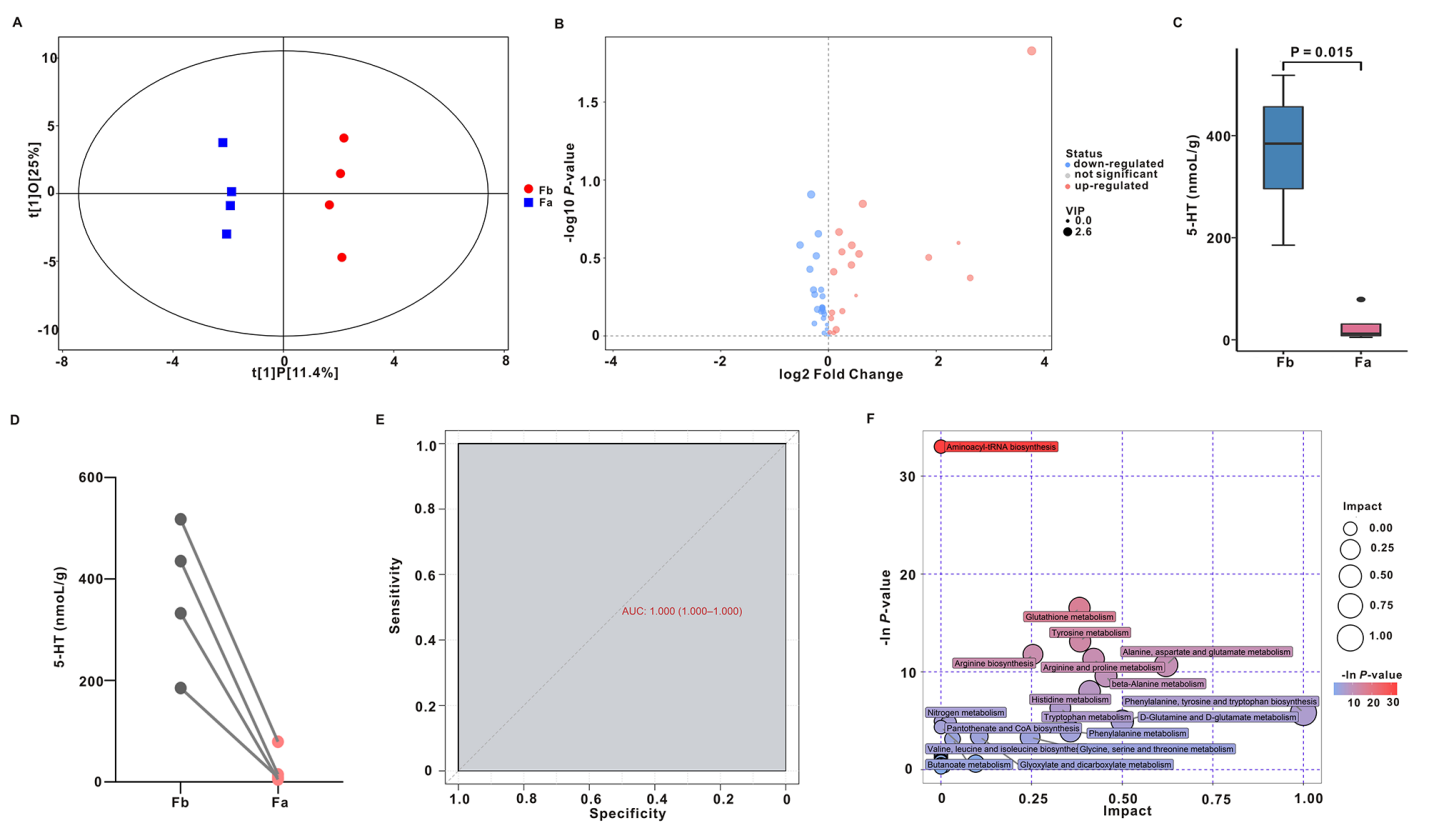
Metabolic patterns before and after FMT in constipation patients with depression. (**A**) Analysis of orthogonal partial least-squares discriminant analysis (OPLS-DA) X-axis representation orthogonal signal correction (OSC) of the score of the main component of the process (t [1]P), Y-axis representation of the score of the OSC process (t [1]O). (**B**) Volcano plot of differential metabolites in the two groups. Blue dots represent downregulation, red dots represent upregulation, and gray indicates non-significant changes. (**C)** 5-HT levels in the two groups. (**D**) Pairwise comparison of 5-HT levels before and after FMT. (**E**) Receiver operating characteristic (ROC) curve of 5-HT in constipation patients with depression. (**F**) Bubble plot of metabolic pathways of differentially abundant metabolites in the two groups

### FMT treatment alleviated the patient gastrointestinal symptoms

All patients reported no adverse events during the FMT process, such as nausea, diarrhea, or infection. Four weeks after FMT, the clinical reaction of stool morphology changed from type II to type IV in all patients. The BFI score in the Fa group (after FMT) was significantly lower than in the Fb group (before FMT) (273.75 ± 30.38 vs. 116.25 ± 110.71, *P* = 0.034). In addition, a significant difference in the KESS score was observed between the Fa and Fb groups (28.25 ± 3.30 vs. 12.25 ± 5.74, *P* = 0.003) (Table 2).

**Table 4:** Differential abundance of metabolites between the Fb and Fa groups

Compound name	Fb_Mean, (nmol/L)	Fa_Mean, (nmol/L)	VIP	*P*-Value	FOLD CHANGE(Fa_mean/Fb_mean)
5-HIAA	47.1935	31.8578	1.4691	0.2977	1.4814
5-HT	368.1041	26.9618	2.5908	0.0149	13.6528
5-HTP	1.9781	1.7923	1.1642	0.9112	1.1037
Ach	539.4731	622.3338	1.0378	0.6755	0.8669
Ala	112548.3949	112959.6761	0.0989	0.9766	0.9964
Arg	12241.0651	13051.2310	0.3198	0.7692	0.9379
Asn	13996.2638	13378.8519	0.6424	0.7094	1.0461
Asp	7188.5534	5327.2456	1.4999	0.2622	1.3494
BAla	905.6876	926.7793	0.0267	0.9061	0.9772
Cys	1308.2768	1587.2374	1.0659	0.5063	0.8242
DA	0.8483	0.8989	0.1708	0.9577	0.9437
DOPAC	12.1566	14.5369	0.9479	0.5418	0.8363
E	6.8521	7.4191	0.5899	0.6703	0.9236
GABA	89.3689	57.5046	1.8181	0.1421	1.5541
GSH	11.2101	3.0888	0.9312	0.3138	3.6293
Gln	112169.8325	114443.4843	0.0226	0.8462	0.9801
Glu	25710.4781	19149.0749	1.3104	0.3507	1.3426
Gly	147517.2019	145217.4144	0.1302	0.9482	1.0158
HVA	6.6168	8.4081	1.1448	0.3735	0.7869
His	29495.5926	31862.7373	0.5170	0.6717	0.9257
Hist	8.4298	9.2744	0.5340	0.6973	0.9089
Kyn	1567.6608	1787.1590	1.3495	0.2212	0.8772
LDOPA	4000.6248	3349.2705	0.5745	0.6931	1.1945
Leu	53691.8125	57996.6485	0.5609	0.5565	0.9258
Lys	27014.8680	26095.7985	0.4207	0.7705	1.0352
Met	11079.1633	12182.5056	0.6242	0.5054	0.9094
NE	7.6155	6.6504	1.5056	0.2156	1.1451
OA	275.4676	344.4639	1.8195	0.1238	0.7997
Orn	11886.2324	12547.9328	0.4288	0.7251	0.9473
Phe	29328.8464	31862.5601	0.4569	0.6538	0.9205
Put	54.9230	79.2411	1.3676	0.2611	0.6931
Ser	23610.6845	22082.6486	1.2432	0.3878	1.0692
Spd	51.4018	36.0744	0.0080	0.5506	1.4249
Spm	1.8376	0.3450	0.0203	0.2532	5.3264
Thr	16967.4760	14281.2878	1.1023	0.2888	1.1881
Trp	28470.6713	30762.5075	0.6639	0.6584	0.9255
TrpA	0.2438	0.2278	0.1484	0.9545	1.0703
Tyr	14325.2015	16764.0158	1.2463	0.3065	0.8545
TyrA	1.3762	1.6511	0.3996	0.8319	0.8335
Val	68949.8420	73676.5301	0.4762	0.6963	0.9358
Melatonin	0.1180	0.0191	0.8390	0.4243	6.1902

5-Hydroxyindoleacetic acid: 5-HIAA, Serotonin: 5-HT, 5-Hydroxytryptophan: 5-HTP, Acetylcholine: Ach, Alanine: Ala, Arginine: Arg, Asparagine: Asn, Aspartate: Asp, β-alanine: BALa, Cysteine: Cys, 3-Hydroxytyramine hydrochloride : DA, 3,4-Dihydroxyphenylacetic acid: DOPAC, Epinephrine: E, 4-Aminobutyric acid: GABA, Glutathione: GSH, Glutamine: Gln, Glutamic acid: Glu, Glycine: Gly, Homovanillic acid: HVA, Histidine: His, Histamine: Hist, Kynurenine: Kyn, 3,4-Dihydroxyphenylalanine: LDOPA, Leucine: Leu, Lysine: Lys, Methionine: Met, Norepinephrine: NE, Octopamine: OA, Ornithine : Orn, Phenylalanine: Phe, Putrescine: Put, Serine: Ser, Spermidine: Spd, Spermine: Spm, Threonine: Thr, Tryptophan: Trp, Tryptamine: TrpA, Tyrosine: Tyr, Tyramine: TyrA, Valine: Val

### FMT treatment alleviated the patient depression and anxiety symptoms

In this study, FMT intervention significantly relieved the anxiety and depression of patients. As shown in Table 2, the HAMD and HAMA scores were significantly lower in the Fa group than Fb group (*P* < 0.001, *P* = 0.006, respectively). Moreover, the SAS and SDS scores were also significantly reduced in Fa group (*P* = 0.020, *P* = 0.047, respectively). In addition, PAC-QOL was used to evaluate the quality of life, and the improvement of the patient’s quality of life could be observed after FMT intervention (*P* = 0.023, Table 2).

**Fig. 4:**
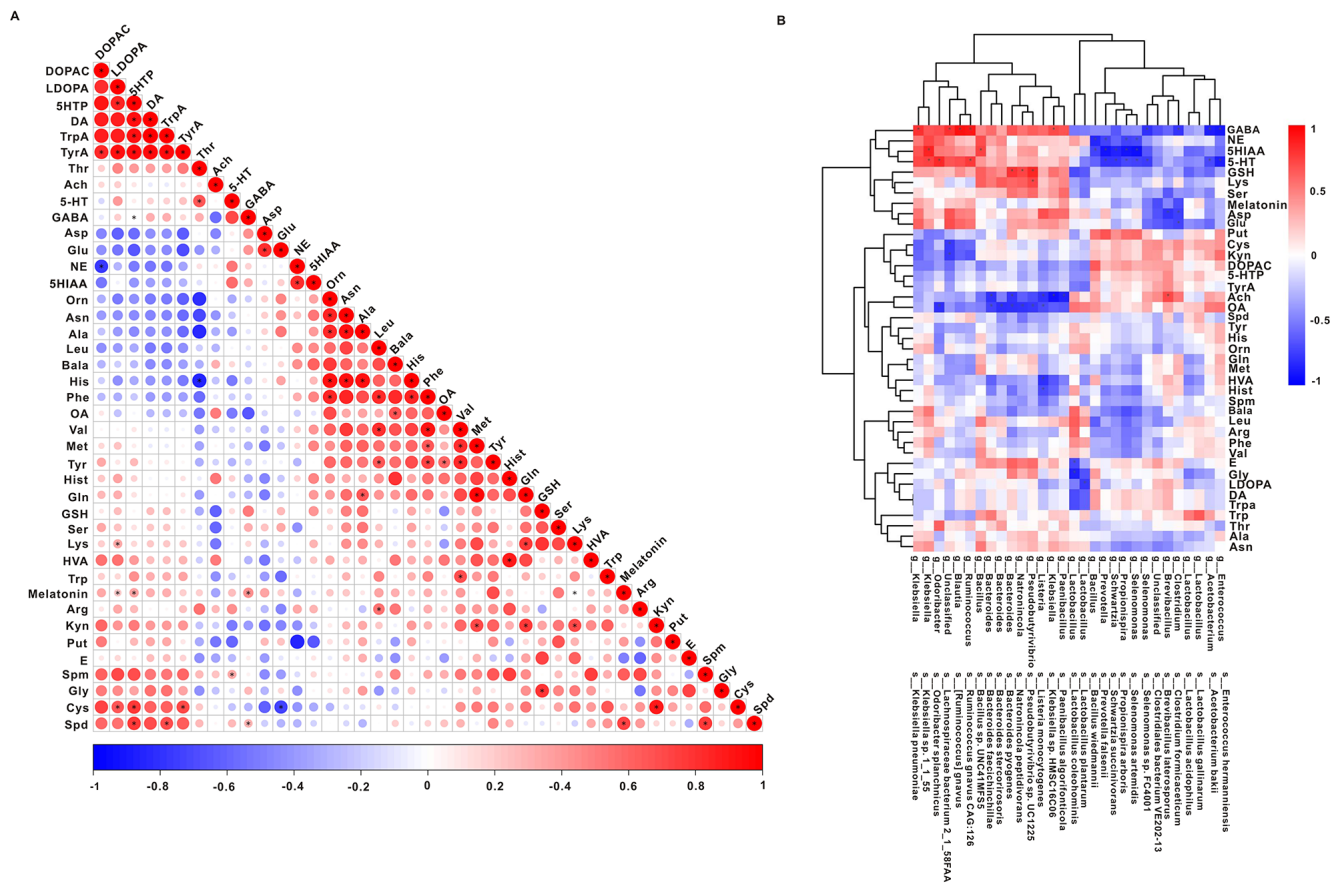
Correlations between metabolites and species. The top 15 different species with upregulated and downregulated expression were detected in metagenomic data. Forty-two targeted neurotransmitter metabolites. (**A**) Correlation analysis between metabolites. (**B**) Correlation analysis between metabolites and different strains

### FMT treatment altered the gene expression and intestinal microbiota composition

The fecal microbiota of 4 donors and 4 patients before and after FMT were analyzed by metagenomic sequencing. The core-pan gene curve was used to evaluate the rationality of sample selection when the curve gradually tended to flatten, indicating that the collected samples meet the requirements of the bioinformatics analysis (Fig. 1A, B). As shown in Fig. 1C, a Venn diagram displayed that 236,480 common genes were generated from the three groups. 39,786 unique genes were identified in the Donor group, 57,813 unique genes in the Fb group, and 47,695 unique genes in the Fa group. To assess the alpha-diversity of the gut microbiota in each group, the following metrics were calculated: chao1, observed_species, and Shannon index. As shown in Fig. 1D, E the chao1 index and observed_species in the Fa group were increased compared to those in the Fb group, but the difference was not statistically significant. Similarly, the Shannon index showed no significant change among the three groups (Fig. 1F).

**Fig. 5:**
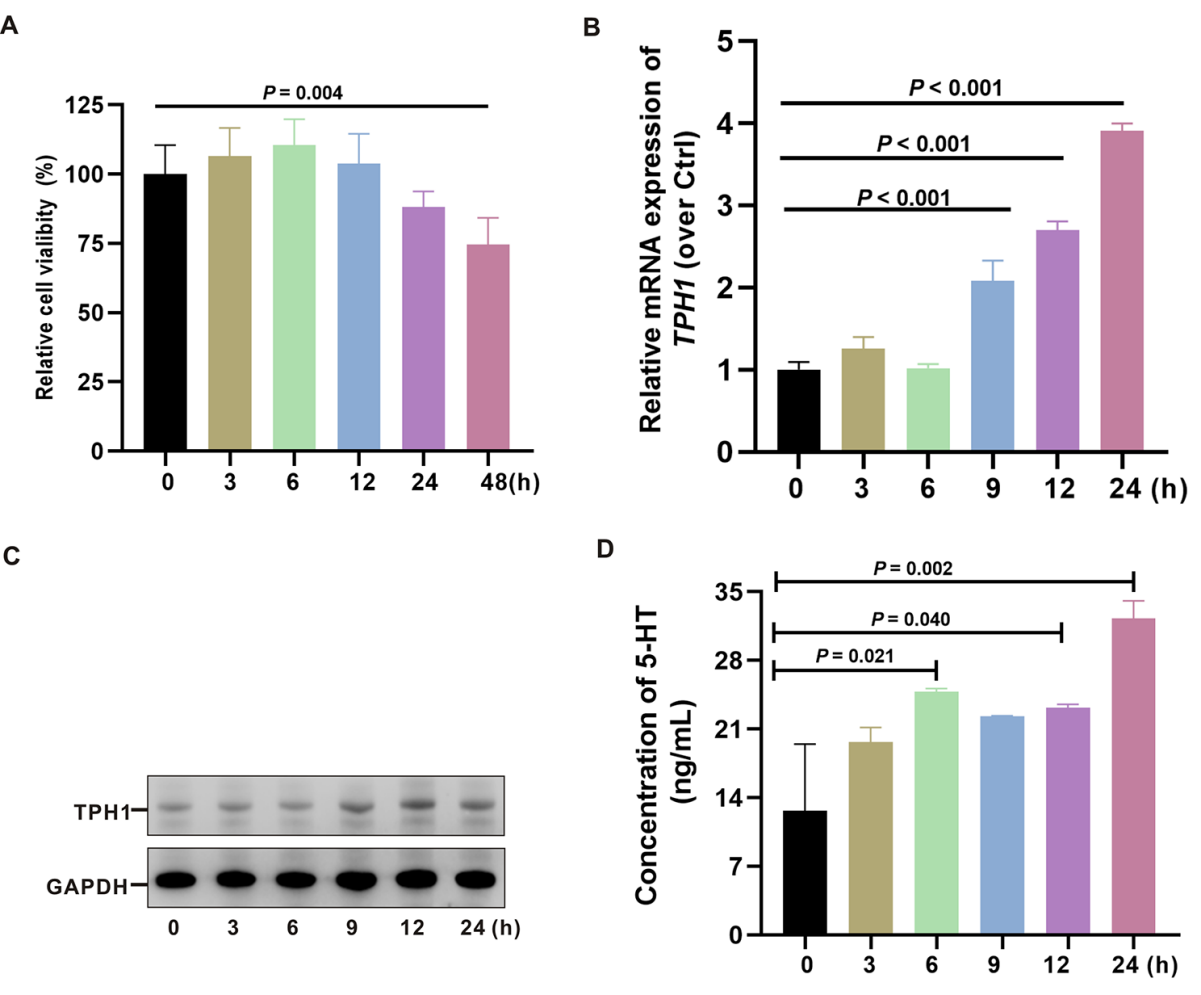
Feces from patients with functional constipation and comorbid psychiatric symptoms increased 5-HT production. (**A**) QGP-1 cells treated with fecal supernatant (fecal supernatant: medium = 1:5) for various times, and cell viability was assessed by CCK-8. (**B, C**) Expression of TPH1 gene and protein as detected using qRT-PCR and WB assay. (**D**) The 5-HT of cell culture medium supernatant was detected by ELISA

Nonmetric multidimensional scaling (NMDS) analysis was used to investigate the comparability of microbial profiles in the three groups. The analysis demonstrated that the bacterial composition of the Fb group was more heterogeneous than Fa group, and the Fa group was more closely related to the Donor group (Fig. 2A). Further, we found that *p_Firmicutes*, *p_Proteobacteria*, *p_Actinobacteria*, and *p_Bacteroidetes* were the main phyla in the three groups (Fig. S1A). At the species level, the top 35 relative abundances were displayed in Fig. 2B and Table S1, revealing that the microbial composition differed in the Fa and Fb groups. Moreover, we found that 141 species demonstrated differential relative abundance between Fa and Fb group (Table S2, Wilcoxon rank-sum test). All the top 15 species with upregulated and downregulated expression are shown in Table 3. Among them, *s_Bacteroides faecichinchillae*, *s_Bacteroides pyogenes*, and *s_Bacteroides stercorirosoris* belonging to *g_Bacteroides* were decreased in the Fa group; *s_Klebsiella pneumoniae*, *s_Klebsiella sp. 1_1_55*, and *s_Klebsiella sp. HMSC16C06* belonging to *g_Klebsiella* were also reduced in the Fa group, whereas *s_Lactobacillus acidophilus*, *s_Lactobacillus coleohominis*, *s_Lactobacillus gallinarum*, and *s_Lactobacillus plantarum* belonging to *g_Lactobacillus* were increased in the Fa group; *s_Selenomonas artemidis* and *s_Selenomonas sp. FC4001* belonging to *g_Selenomona*s were also increased in the Fa group.

To understand the role of vital species in the pathogenesis of constipation in patients with depression and anxiety, we used residents and colonizers to identify critical species. Ruiqiao et al. first divided the intestinal bacteria among recipients after FMT into residents and colonizers [24]. Residents are bacteria with high abundance in patients before FMT, whereas colonizers are bacteria with low abundance or absence in patients before FMT. We found three residents (*s_Bacteroides faecichinchillae*, *s_Bacteroides pyogenes*, and *s_Bacteroides stercorirosoris*) and three colonizers (*s_Brevibacillus laterosporus*, *s_Enterococcus hermanniensis*, and *s_Clostridiales bacterium VE202-13*) (Table 3). Furthermore, paired analysis before and after FMT revealed that *s_Enterococcus hermanniensis* and *s_Bacteroides pyogenes* remained constant in the four samples (Fig. 2C-F).

### FMT treatment altered the serum metabolite profile

Microbiota-derived metabolites affect the host through a variety of signaling pathways. Increasing evidence has shown that some metabolites of the gut microbiota can enter the bloodstream and exhibit vital influences on the mental and behavioural health of the host [25]. Hence, UHPLC–MS/MS was used to analyze the host metabolic profile of neurotransmitters. The serum samples before and after FMT were predominantly separated according to orthogonal partial least squares discriminant analysis (OPLS-DA) (Fig. 3A) and volcano plots (Fig. 3B). Of the 42 neurotransmitters, 5-HT was decreased significantly in the Fa (26.96 nmol/L) group compared to the Fb (368.10 nmol/L) group (Fig. 3C, D; Table 4, P = 0.015). Serum 5-HT level was used as a variable test, and whether belonging to Fa or Fb group to draw the ROC curve. The results showed that the AUC was 1.00 (Fig. 3E), suggesting that the 5-HT level was related to FMT. Furthermore, the levels of Trp, tryptamine (TrpA), 5-hydroxyindolecetic acid (5-HIAA), 5-hydroxytryptophan (5-HTP), and kynurenine (Kyn), which are related to 5-HT metabolism, were not significantly different in Fa compared to Fb (Fig. S2). The 33 KEGG pathways significantly differed between the two groups (Fig. 3F). The top 3 enrichment pathways included aminoacyl-tRNA biosynthesis, glutathione metabolism, and tyrosine metabolism.

The Spearman correlation method was used to generate a correlation matrix to explore the potential relationships between different metabolites and the gut microbiome. The level of 5-HT was positively correlated with the level of threonine (R = 0.643, *P* = 0.47, Fig. 4A). The abundance of most species, including *s_Klebsiella sp. 1_1_55*, *s_Odoribacter splanchnicus*, and s*_Ruminococcus gnavus CAG:12*6, were positively correlated with the level of 5-HT, *s_Acetobacterium bakii*, *s_Enterococcus hermanniensis*, *s_Prevotella falsenii*, *s_Propionispira arboris*, *s_Schwartzia succinivorans*, *s_Selenomonas artemidis*, and *s_Selenomonas sp. FC4001* was negatively correlated with the level of 5-HT (Fig. 4B).

### Feces from patients with functional constipation and comorbid psychiatric symptoms increased 5-HT production

To explore the potential relationship between elevated serum 5-HT levels and gut microbiota metabolites, the QGP-1 cells, a well-established neuroendocrine enterochromaffin cell line for studying 5-HT production, were treated with fecal supernatant from patients to establish an exposed model in vitro. No significant cytotoxicity was observed with fecal supernatant exposure (Fig. 5A). Tryptophan hydroxylase 1 (TPH-1) is the key rate-limiting enzyme for peripheral 5-HT synthesis. The relative expression levels of the TPH-1 gene and protein were increased in QGP-1 cells exposed to patient fecal supernatant (fecal supernatant to medium 1:5) (Fig. 5B, C). Similarly, elevated 5-HT was observed in cell culture supernatants exposed to patient fecal supernatants compared to controls (Fig. 5D). These results suggest that 5-HT alterations are associated with intestinal metabolites.

## Discussion

FMT is a widely used approach to remodel the gut microbiota. Our findings revealed that symptoms of constipation were relieved by FMT, consistent with previous studies. The depression-relieving effect of FMT was also confirmed, and the HAMD, HAMA, SDS, and SAS scores were significantly different before and after FMT. Similarly, a case report study indicated that depression symptoms improved significantly 4 weeks after FMT in two patients with major depressive disorder [26]. Furthermore, a previous study indicated that depression and anxiety symptoms might be alleviated by FMT in patients with IBS, functional diarrhea, or functional constipation, and a correlation was noted between microbiota diversity and HAMD [20]. Nonetheless, our study’s constraint was limited sample size. Thus, in the future, large sample size, double-blind, randomized, placebo-controlled trials are needed to comprehensively investigate the effects and mechanisms of FMT in relieving functional constipation combined with psychiatric symptoms.

The intestinal microbiota is vital to host health and has recently become the target of living bacterial cell biological therapy for numerous chronic diseases, such as chronic constipation and depression. In this study, many taxa exhibited differential relative abundance before and after FMT as assessed by metagenomics sequencing. Compared with before transplantation, the relative abundance of *g_Bacteroides* and *g_Klebsiella* was significantly decreased after FMT intervention for 4 weeks, whereas the relative abundance of *g_Lactobacillus* and *g_Selenomonas* was significantly increased. In our previous study, *g_Bacteroidetes* were more abundant in the fecal microbiota of constipated women of reproductive age [27]. Similarly, compared with the control, the abundance of *g_Bacteroides* was 1.5 times higher in the mucosal microbiota of constipation patients [28, 29]. Furthermore, the random forest algorithm confirmed that the relative abundance of *g_Bacteroidetes* was inversely correlated with colonic transit [28]. These studies suggest that *g_Bacteroidetes* may be involved in constipation. Interestingly, Wu et al. found that *Bacteroidetes* were negatively correlated with dietary fiber intake [30]. Tian et al. found that the high abundance of *Bacteroides* and *Klebsiella* bacteria in feces may cause constipation, and their relative levels decreased after FMT [31]. However, fecal qRT‒PCR results showed that the relative abundance of *Bifidobacterium* and *Bacteroides* species in stool samples from constipated patients was significantly lower than that of healthy controls [32]. Thus, we speculate that this may be due to different detection methods and disease subtypes. In addition, lower *g-Lactobacillus* levels were found in chronic functional constipation [31, 33] and IBS subjects [34]. Consistent with our research results, Lulu Xie et al. found that the relative abundance of *g_Lactobacillus* was higher after FMT intervention than at baseline [35]. *Lactobacillus paracei* alleviates constipation symptoms by increasing the level of short-chain fatty acids and promoting intestinal motility [36]. *Lactobacillus acidophilus* promotes intestinal electrolyte absorption by increasing Cl^−^/HCO^3−^ and Na^+^/H^+^ transport [37, 38]. In addition, *Lactobacillus* can relieve stress-induced anxiety and depression-related behaviour and regulate central γ-aminobutyric acid receptor expression [39]. Zhou Dan et al. implicated that the decreased levels of *Bacteroides spp.* and *Prevotella spp.* may cause abnormal dopamine signaling by regulating amino acid metabolism in autism spectrum disorder patients [40]. An experimental animal study showed that the improvement of depression-like behavior by FMT may be associated with an increase in 5-HT levels and decreases in IL-1β and TNF-α levels [41]. Thus, FMT intervention increased the bacteria associated with intestinal motility, such as *g_Lactobacillus*, and decreased the bacteria associated with psychiatric illness, such as *g_Bacteroides*, to relieve constipation and psychiatric symptoms.

5-HT is a common inhibitory neurotransmitter in the central nervous system and enteric nervous system. Previously, studies have shown that the altered 5-HT signal pattern leads to increased 5-HT content, 5-HT release, and enterochromaffin cell numbers but does not involve a decrease in serotonin selective reuptake transporter (SERT) expression [42, 43]. Similarly, in this study, the 5-HT level was significantly increased at baseline but subsequently decreased 13-fold after FMT intervention for 4 weeks in patients with constipation and psychiatric symptoms (Fig. 3). Moreover, serotonin signaling has also been studied in many animal models, including TNBS colitis [44], DSS colitis [45], and *Trichinella spiralis* enteritis in mice [46]. In all these conditions, the level of 5-HT, the release of 5-HT, and the number of enterochromaffin cells were increased. Another consistent feature of these models was the reduction in epithelial SERT expression. It has been demonstrated that this reduction in SERT levels leads to increased availability of 5-HT under basic and stimulus conditions [47]. In addition, Narek et al. found reductions in the amount of 5-HT released by intestinal neurons, which led to deficiencies in enteric nervous system development and gastrointestinal motility in the TPH2-R439H mouse model [48]. Furthermore, we found that fecal supernatants from patients with constipation combined with depression and anxiety promoted 5-HT synthesis and secretion in QGP-1 cells. More interestingly, we observed the same effect in *Bacteroides* supernatants exposed to QGP-1 cells. The findings suggest that 5-HT links constipation with emotional disorders and that specific bacteria may play a key role. Although increasing evidence supports the concept that 5-HT signaling is altered in functional gastrointestinal diseases and mental disorders, however, its causal and effective relationship still needs to be resolved.

Although our study provided some clues for FMT treatment of constipation patients with psychiatric symptoms, many limitations still exist. First, this study included a small sample with no placebo control. Second, we only performed serum metabolomic sequencing, and if both serum and fecal samples were metabolomic sequenced, it would provide a more comprehensive understanding of the disease. Fourth, although we performed a combined multiomics analysis, the association between different strains and changes in 5-HT content still needs to be explored. Third, the molecular regulatory mechanism of significantly reduced 5-HT levels after FMT intervention remains unclear.

## Conclusions

After 4 weeks of FMT intervention, constipation, depression, and anxiety symptoms were significantly alleviated in the participants. FMT intervention altered the gut microbiota profile at the phylum, genus, and species levels. The 5-HT content was significantly diminished after FMT intervention compared with baseline. Additionally, the patient’s fecal supernatant demonstrated an ability to enhance 5-HT biosynthesis in vitro. Further studies are required to investigate the relationship between gut microbiome-mediated metabolites and neural function. Taken together, our study provided valuable insights into the connection among gut microbiota, metabolites, intestinal dysfunction, and neurotransmitter dysregulation in constipated patients with psychiatric symptoms.

### Electronic supplementary material

Below is the link to the electronic supplementary material.

Supplementary Material 1

Supplementary Material 2

Supplementary Material 3

Supplementary Material 4

Supplementary Material 5

## Data Availability

The datasets presented in this study can be found in online repositories. The names of the repository and accession number can be found below: NCBI PRJNA909986.

## References

[1] Barberio B, Judge C, Savarino EV, Ford AC (2021). Global prevalence of functional constipation according to the Rome criteria: a systematic review and meta-analysis. Lancet Gastroenterol Hepatol.

[2] Suares NC, Ford AC. Prevalence of, and risk factors for, chronic idiopathic constipation in the community: systematic review and meta-analysis. Am J Gastroenterol 2011, 106(9). 10.1038/ajg.2011.164 21606976

[3] Camilleri M, Ford AC, Mawe GM, Dinning PG, Rao SS, Chey WD (2017). Chronic constipation. Nat Rev Dis Primers.

[4] Fond G, Loundou A, Hamdani N, Boukouaci W, Dargel A, Oliveira J (2014). Anxiety and depression comorbidities in irritable bowel syndrome (IBS): a systematic review and meta-analysis. Eur Arch Psychiatry Clin Neurosci.

[5] Mykletun A, Jacka F, Williams L, Pasco J, Henry M, Nicholson GC (2010). Prevalence of mood and anxiety disorder in self reported irritable bowel syndrome (IBS). An epidemiological population based study of women. BMC Gastroenterol.

[6] Fuller-Thomson E, Sulman J (2006). Depression and inflammatory bowel Disease: findings from two nationally representative Canadian surveys. Inflamm Bowel Dis.

[7] Bhandari S, Larson ME, Kumar N, Stein D (2017). Association of Inflammatory Bowel Disease (IBD) with depressive symptoms in the United States Population and Independent predictors of depressive symptoms in an IBD Population: a NHANES Study. Gut Liver.

[8] Singh P, Agnihotri A, Pathak MK, Shirazi A, Tiwari RP, Sreenivas V (2012). Psychiatric, somatic and other functional gastrointestinal disorders in patients with irritable bowel syndrome at a tertiary care center. J Neurogastroenterol Motil.

[9] Labus JS, Hollister EB, Jacobs J, Kirbach K, Oezguen N, Gupta A (2017). Differences in gut microbial composition correlate with regional brain volumes in irritable bowel syndrome. Microbiome.

[10] Labus JS, Oezguen N, Hollister EB, Tillisch K, Savidge T, Versalovic J et al. 752 Regional Brain morphology is Associated with Gut Microbial metabolites in Irritable Bowel Syndrome (IBS). 2015, 148(4):S–142.

[11] Tillisch K, Labus J, Kilpatrick L, Jiang Z, Stains J, Ebrat B et al. Consumption of fermented milk product with probiotic modulates brain activity. Gastroenterology 2013, 144(7). 10.1053/j.gastro.2013.02.043 PMC3839572 23474283

[12] Zhang X, Chen S, Zhang M, Ren F, Ren Y, Li Y et al. Effects of fermented milk containing strain Shirota on Constipation in patients with Depression: a Randomized, Double-Blind, placebo-controlled trial. Nutrients 2021, 13(7). 10.3390/nu13072238 PMC8308326 34209804

[13] Fan Y, Pedersen O (2021). Gut microbiota in human metabolic health and Disease. Nat Rev Microbiol.

[14] Tian H, Ding C, Gong J, Ge X, McFarland LV, Gu L (2016). Treatment of slow transit constipation with fecal microbiota transplantation: a pilot study. J Clin Gastroenterol.

[15] Huang HL, Chen HT, Luo QL, Xu HM, He J, Li YQ (2019). Relief of irritable bowel syndrome by fecal microbiota transplantation is associated with changes in diversity and composition of the gut microbiota. J Dig Dis.

[16] Weingarden AR, Vaughn BP (2017). Intestinal microbiota, fecal microbiota transplantation, and inflammatory bowel Disease. Gut Microbes.

[17] Langgartner D, Vaihinger CA, Haffner-Luntzer M, Kunze JF, Weiss A-LJ, Foertsch S (2018). The role of the intestinal microbiome in chronic psychosocial stress-Induced pathologies in male mice. Front Behav Neurosci.

[18] Yang C, Fang X, Zhan G, Huang N, Li S, Bi J (2019). Key role of gut microbiota in anhedonia-like phenotype in rodents with neuropathic pain. Transl Psychiatry.

[19] Mizuno S, Masaoka T, Naganuma M, Kishimoto T, Kitazawa M, Kurokawa S (2017). Bifidobacterium-Rich Fecal Donor May be a positive predictor for successful fecal microbiota transplantation in patients with irritable bowel syndrome. Digestion.

[20] Kurokawa S, Kishimoto T, Mizuno S, Masaoka T, Naganuma M, Liang K-C (2018). The effect of fecal microbiota transplantation on psychiatric symptoms among patients with irritable bowel syndrome, functional diarrhea and functional constipation: an open-label observational study. J Affect Disord.

[21] Drossman DA. Functional Gastrointestinal Disorders: History, Pathophysiology, Clinical Features and Rome IV. Gastroenterology 2016. 10.1053/j.gastro.2016.02.032 27144617

[22] Yang C, Bai X, Hu T, Xue X, Su X, Zhang X (2022). Integrated metagenomics and targeted-metabolomics analysis of the effects of phenylalanine on loperamide-induced constipation in rats. Front Microbiol.

[23] Alba AC, Agoritsas T, Walsh M, Hanna S, Iorio A, Devereaux PJ (2017). Discrimination and calibration of clinical prediction models: users’ guides to the Medical Literature. JAMA.

[24] He R, Li P, Wang J, Cui B, Zhang F, Zhao F (2022). The interplay of gut microbiota between donors and recipients determines the efficacy of fecal microbiota transplantation. Gut Microbes.

[25] Rutsch A, Kantsjö JB, Ronchi F (2020). The gut-brain Axis: how microbiota and host Inflammasome Influence Brain Physiology and Pathology. Front Immunol.

[26] Doll JPK, Vázquez-Castellanos JF, Schaub A-C, Schweinfurth N, Kettelhack C, Schneider E (2022). Fecal microbiota transplantation (FMT) as an adjunctive therapy for Depression-Case Report. Front Psychiatry.

[27] Li H, Chen J, Ren X, Yang C, Liu S, Bai X (2020). Gut microbiota composition changes in Constipated women of Reproductive Age. Front Cell Infect Microbiol.

[28] Parthasarathy G, Chen J, Chen X, Chia N, O’Connor HM, Wolf PG et al. Relationship between microbiota of the Colonic Mucosa vs feces and symptoms, Colonic Transit, and methane production in female patients with chronic constipation. Gastroenterology 2016, 150(2). 10.1053/j.gastro.2015.10.005 PMC4727996 26460205

[29] Parkes GC, Rayment NB, Hudspith BN, Petrovska L, Lomer MC, Brostoff J (2012). Distinct microbial populations exist in the mucosa-associated microbiota of sub-groups of irritable bowel syndrome. Neurogastroenterol Motil.

[30] Wu GD, Chen J, Hoffmann C, Bittinger K, Chen Y-Y, Keilbaugh SA (2011). Linking long-term dietary patterns with gut microbial enterotypes. Science.

[31] Tian Y, Zuo L, Guo Q, Li J, Hu Z, Zhao K (2020). Potential role of fecal microbiota in patients with constipation. Th Adv Gastroenterol.

[32] Kim SE, Choi SC, Park KS, Park MI, Shin JE, Lee TH (2015). Change of Fecal Flora and Effectiveness of the short-term VSL#3 Probiotic treatment in patients with functional constipation. J Neurogastroenterol Motil.

[33] Khalif IL, Quigley EMM, Konovitch EA, Maximova ID (2005). Alterations in the colonic flora and intestinal permeability and evidence of immune activation in chronic constipation. Dig Liver Dis.

[34] Pimentel M, Lembo A (2020). Microbiome and its role in irritable bowel syndrome. Dig Dis Sci.

[35] Xie L, Xu C, Fan Y, Li Y, Wang Y, Zhang X (2021). Effect of fecal microbiota transplantation in patients with slow transit constipation and the relative mechanisms based on the protein digestion and absorption pathway. J Transl Med.

[36] Zhuang M, Shang W, Ma Q, Strappe P, Zhou Z (2019). Abundance of Probiotics and Butyrate-Production Microbiome manages constipation via short-chain fatty acids production and hormones secretion. Mol Nutr Food Res.

[37] Raheja G, Singh V, Ma K, Boumendjel R, Borthakur A, Gill RK (2010). Lactobacillus acidophilus stimulates the expression of SLC26A3 via a transcriptional mechanism. Am J Physiol Gastrointest Liver Physiol.

[38] Singh V, Raheja G, Borthakur A, Kumar A, Gill RK, Alakkam A (2012). Lactobacillus acidophilus upregulates intestinal NHE3 expression and function. Am J Physiol Gastrointest Liver Physiol.

[39] Bravo JA, Forsythe P, Chew MV, Escaravage E, Savignac HM, Dinan TG (2011). Ingestion of Lactobacillus strain regulates emotional behavior and central GABA receptor expression in a mouse via the vagus nerve. Proc Natl Acad Sci U S A.

[40] Dan Z, Mao X, Liu Q, Guo M, Zhuang Y, Liu Z (2020). Altered gut microbial profile is associated with abnormal metabolism activity of Autism Spectrum Disorder. Gut Microbes.

[41] Rao J, Qiao Y, Xie R, Lin L, Jiang J, Wang C (2021). Fecal microbiota transplantation ameliorates stress-induced depression-like behaviors associated with the inhibition of glial and NLRP3 inflammasome in rat brain. J Psychiatr Res.

[42] Costedio MM, Coates MD, Brooks EM, Glass LM, Ganguly EK, Blaszyk H (2010). Mucosal serotonin signaling is altered in chronic constipation but not in opiate-induced constipation. Am J Gastroenterol.

[43] Lincoln J, Crowe R, Kamm MA, Burnstock G, Lennard-Jones JE (1990). Serotonin and 5-hydroxyindoleacetic acid are increased in the sigmoid colon in severe idiopathic constipation. Gastroenterology.

[44] Linden DR, Foley KF, McQuoid C, Simpson J, Sharkey KA, Mawe GM (2005). Serotonin transporter function and expression are reduced in mice with TNBS-induced Colitis. Neurogastroenterol Motil.

[45] O’Hara JR, Skinn AC, MacNaughton WK, Sherman PM, Sharkey KA (2006). Consequences of Citrobacter rodentium Infection on enteroendocrine cells and the enteric nervous system in the mouse colon. Cell Microbiol.

[46] Wheatcroft J, Wakelin D, Smith A, Mahoney CR, Mawe G, Spiller R (2005). Enterochromaffin cell hyperplasia and decreased serotonin transporter in a mouse model of postinfectious bowel dysfunction. Neurogastroenterol Motil.

[47] Linden DR, Chen J-X, Gershon MD, Sharkey KA, Mawe GM (2003). Serotonin availability is increased in mucosa of guinea pigs with TNBS-induced Colitis. Am J Physiol Gastrointest Liver Physiol.

[48] Israelyan N, Del Colle A, Li Z, Park Y, Xing A, Jacobsen JPR et al. Effects of Serotonin and slow-release 5-Hydroxytryptophan on gastrointestinal motility in a mouse model of Depression. Gastroenterology 2019, 157(2). 10.1053/j.gastro.2019.04.022 PMC6650329 31071306

