# Supplementary figures and images for: Multi-omics analysis of fecal microbiota transplantation’s impact on functional constipation and comorbid depression and anxiety
Source: BMC Microbiol. 2023 Dec 7;23:389. doi: 10.1186/s12866-023-03123-1 (PMC10701952; doi:10.1186/s12866-023-03123-1)

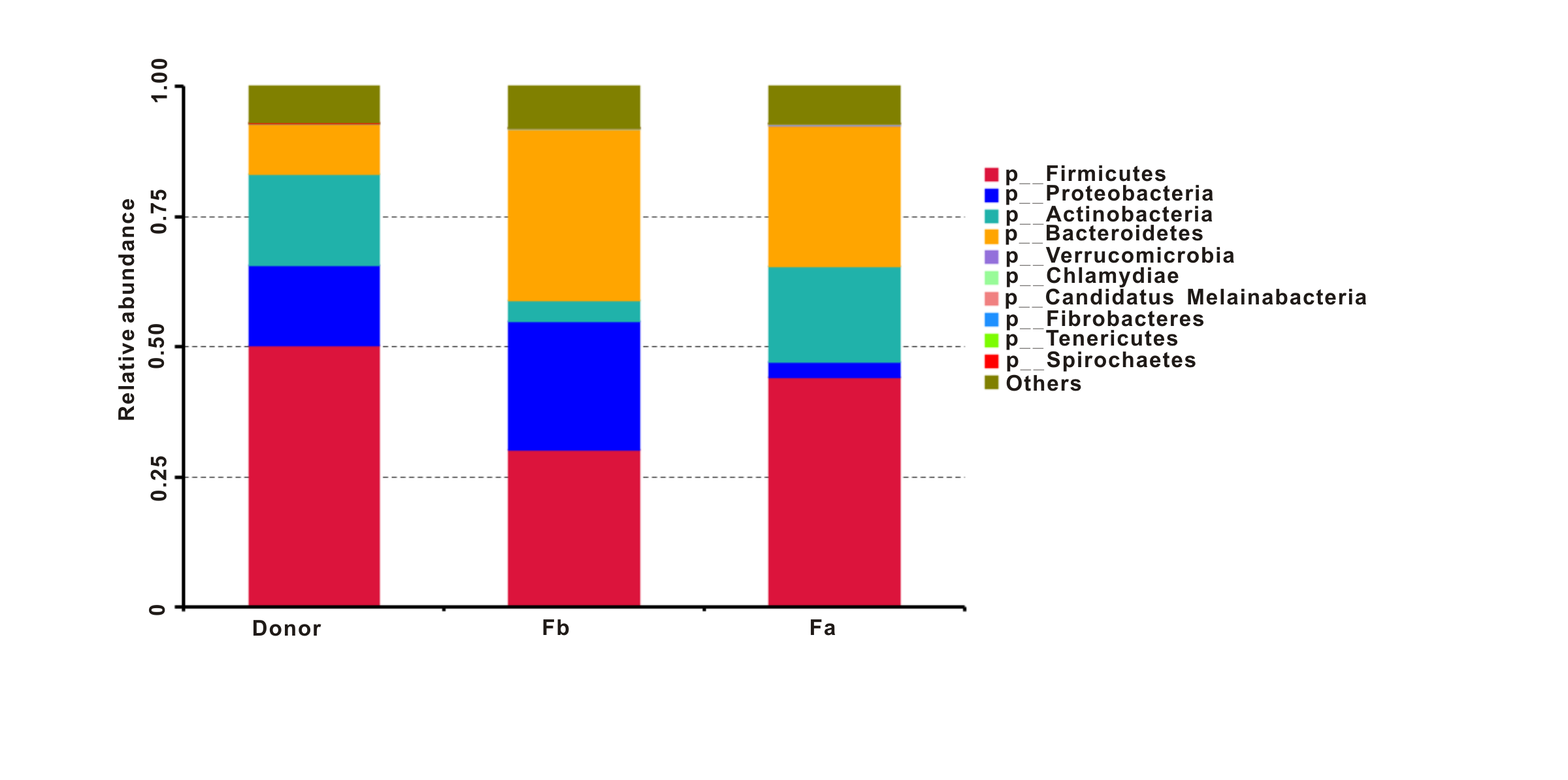

Supplement: Supplementary file 1 — Supplementary Material 1 [file 12866_2023_3123_MOESM1_ESM.tif]

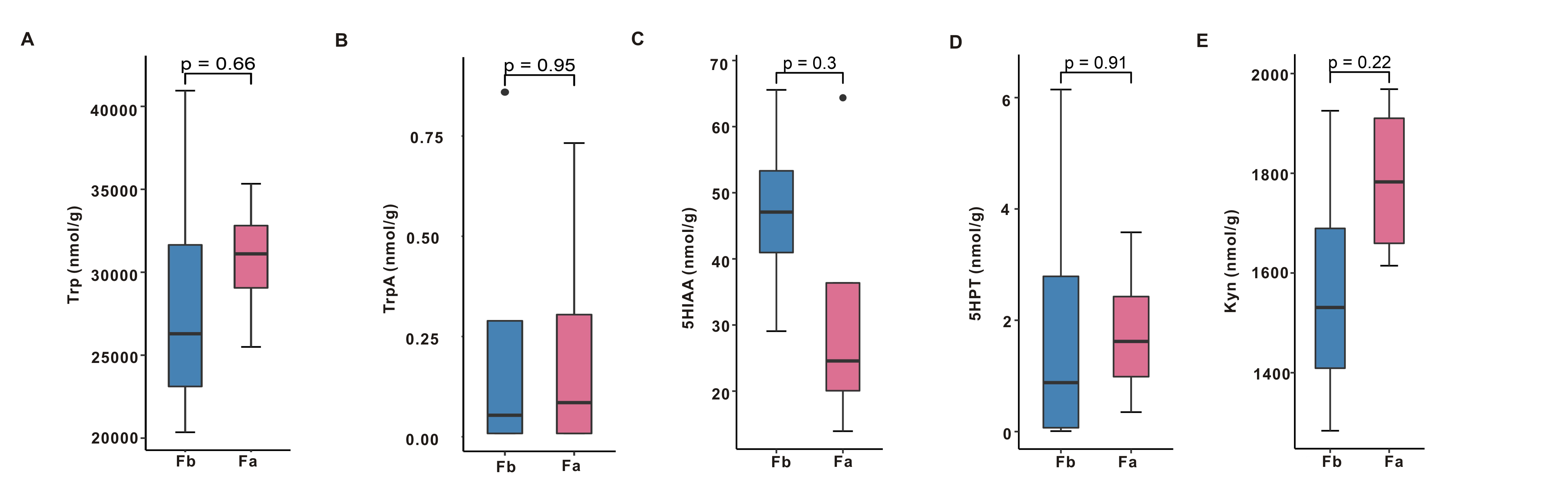

Supplement: Supplementary file 2 — Supplementary Material 2 [file 12866_2023_3123_MOESM2_ESM.tif]
