# Supplementary material for: Multi-omics analysis of fecal microbiota transplantation’s impact on functional constipation and comorbid depression and anxiety
Source: BMC Microbiol. 2023 Dec 7;23:389. doi: 10.1186/s12866-023-03123-1 (PMC10701952; doi:10.1186/s12866-023-03123-1)

**Fig. S1** Top 10 gut microbiome compositions in the three groups at the phylum

**Fig. S2** Box plot of metabolites across the Fa and Fb groups. (**A**) Tryptophan (Trp), (**B**) tryptamine (TrpA), (**C**) 5-hydroxyindolecetic acid (5HIAA), (**D**) 5-hydroxytryptophan (5-HTP) and (**E**) kynurenine (Kyn).

**Table S1** The top 35 different species in the three groups.

**Table S2** Differential relative abundance of 141 species between the Fb and Fa groups.

original images of full-length


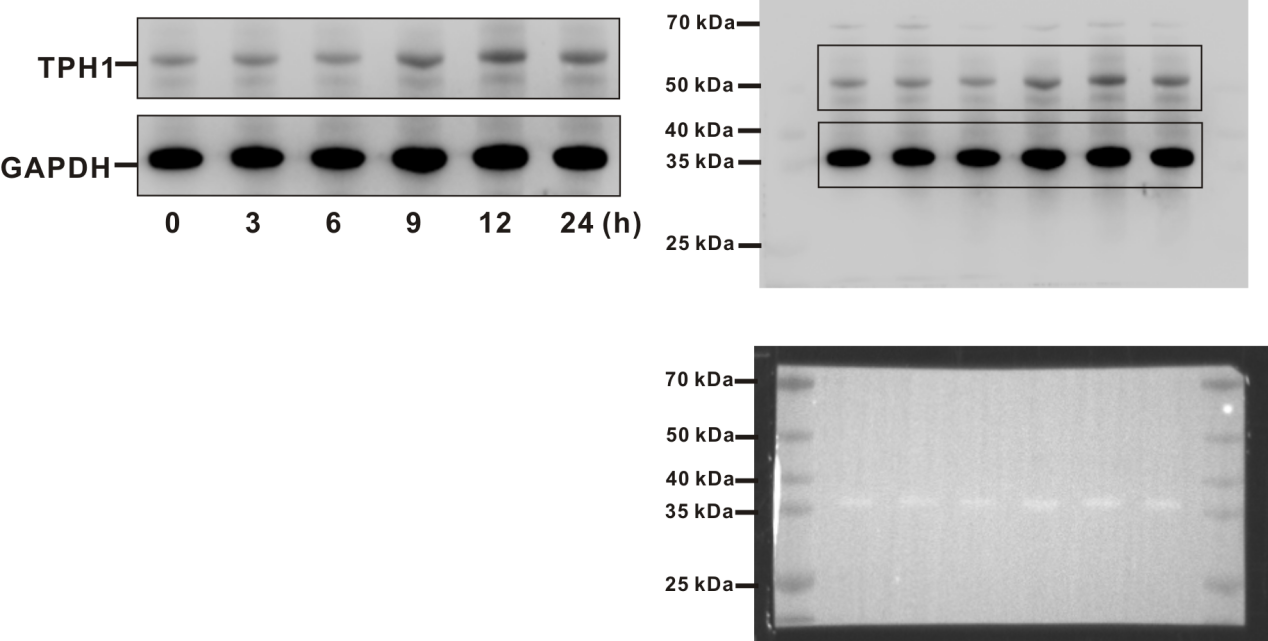

Supplement: Supplementary file 5 — Supplementary Material 5 [file 12866_2023_3123_MOESM5_ESM.docx]
